# Probiotics in epilepsy treatment: a meta-analysis of efficacy and safety

**DOI:** 10.3389/fmicb.2025.1676480

**Published:** 2025-12-11

**Authors:** Kang Tan, Yunfeng Yu, Pei Liu, Pengfei Jiang, Yi Liu, Bidan Lou, Qinghua Peng

**Affiliations:** 1 School of Traditional Chinese Medicine, Hunan University of Chinese Medicine, Changsha, China; 2 Acupuncture-Tuina-Rehabilitation Department, The First Hospital of Hunan University of Chinese Medicine, Changsha, China; 3 Quzhou Hospital Ophthalmology Center, Zhejiang Medical and Health University, Quzhou, China

**Keywords:** probiotics, epilepsy, antiepileptic drugs, systematic review, meta-analysis

## Abstract

**Background:**

Interest in probiotics for managing neurological disorders has grown recently.

**Methods:**

Eight public databases were searched for relevant randomized controlled trials (RCTs) published until May 31, 2025. Eligible studies were screened based on predefined inclusion and exclusion criteria. Basic characteristics and outcome data were extracted from the included studies, and the risk of bias was assessed. Meta-analyses, sensitivity analyses, and publication bias evaluations were performed using RevMan 5.3 software. The quality of evidence was rated using GRADEpro GDT.

**Results:**

Six RCTs involving 656 patients were included. Compared with the AED group, the probiotic combination group had significantly higher clinical efficacy rate (relative risk [RR] 1.16, 95% confidence interval [CI] 1.09 to 1.24, *p* < 0.00001) and lower seizure frequency (mean difference [MD] −1.97, 95% CI −2.35 to −1.59, *p* < 0.00001), seizure duration (MD −0.56, 95% CI −0.74 to −0.38, *p* < 0.00001), electroencephalogram (EEG) *θ* band relative power (MD −1.89, 95% CI −2.51 to −1.26, *p* < 0.00001), serum diamine oxidase (DAO) levels (MD −1.26, 95% CI −1.61 to −0.90, *p* < 0.00001), and serum D-lactate (D-LAC) levels (MD −2.04, 95% CI −2.78 to −1.30, *p* < 0.00001). No statistically significant differences were observed between the two groups in terms of interictal epileptiform discharge (MD −1.82, 95% CI −4.04 to 0.41, *p* = 0.11), EEG *δ* band relative power (MD 0.13, 95% CI −0.23 to 0.48, *p* = 0.49), EEG *α* band relative power (MD −0.27, 95% CI −1.34 to 0.80, *p* = 0.62), and EEG *β* band relative power (MD 0.10, 95% CI −0.17 to 0.38, *p* = 0.47). No significant difference was observed in the total adverse reaction rate between two groups (RR 0.58, 95% CI 0.32 to 1.08, *p* = 0.08). Funnel plots revealed no publication bias for clinical efficacy rate, seizure frequency, seizure duration, EEG *β* band relative power, and serum DAO and D-LAC levels.

**Conclusion:**

Probiotics improved seizure activity, relative power in certain EEG frequency bands, and intestinal barrier function without increasing the incidence of adverse reactions, supporting their feasibility as complementary treatment for epilepsy. Limited sample size and methodological necessitate further validation.

**Systematic review registration:**

https://www.crd.york.ac.uk/PROSPERO/view/CRD420251079172, Identifier CRD420251079172.

## 1 Introduction

Epilepsy is a common neurological disorder characterized by recurrent epileptiform discharges that result in temporary neurological dysfunction (Krishnamurthy, 2025). According to the World Health Organization, approximately 50 million people worldwide have epilepsy (Mwanga et al., 2024). Epilepsy is characterized by a high incidence rate, therapeutic recalcitrance, and substantial comorbidity burden, placing a heavy burden on patients, families, and society (Keezer, 2025). Current clinical management strategies for epilepsy include antiepileptic drugs (AEDs), surgical intervention, neuromodulation therapy, and the ketogenic diet (Li et al., 2025). Although these treatments can be used to control seizures, they have significant limitations. Approximately 30% of cases of epilepsy remain refractory to adequate AED regimens and progress to drug-resistant epilepsy (DRE) (Klein et al., 2024). Long-term administration of AEDs may induce adverse effects such as somnolence, dizziness, and cognitive impairment, markedly compromising the patients’ quality of life (Li et al., 2025). Surgical interventions have narrow applicability and carry inherent perioperative risks (Kolosky et al., 2024), neuromodulation devices are costly (Salama et al., 2024), and the ketogenic diet is associated with low patient adherence (Hameed et al., 2025). Therefore, development of safe and effective novel therapeutic strategies for epilepsy is an urgent priority.

Recent advancements in the “brain-gut axis” theory have revealed new therapeutic possibilities for epilepsy. This theory posits that the gut microbiota and the central nervous system are connected by a bidirectional regulatory network through which dynamic communication occurs via microbial metabolites, immune modulation, and neuroendocrine pathways (Yue et al., 2022). Several clinical studies have revealed notable differences in the composition of gut microbiota between individuals with epilepsy and healthy individuals (Gong et al., 2020; Türay et al., 2023; Wei et al., 2023). These differences are characterized by a reduced abundance of beneficial bacteria such as *Bifidobacterium* and *Lactobacilli*, an increased presence of opportunistic pathogens such as *Escherichia coli*, and diminished microbial diversity (Zhu et al., 2024). This dysbiosis is particularly pronounced in patients with DRE (Chen et al., 2024). Mechanistic investigations have revealed that gut microbial metabolites contribute to epileptogenesis by regulating neurotransmitter synthesis, disrupting intestinal barrier integrity, and activating inflammatory responses (Zhu et al., 2024). Preclinical studies have demonstrated that gut microbiota dysbiosis impairs blood–brain barrier integrity, activates microglia and promotes the release of proinflammatory cytokines such as IL-1, IL-6, IL-17, TNF-α, and LPS (Gong et al., 2024). This lowers the seizure threshold, exacerbates disease progression, and prolongs seizure duration (Kew et al., 2020). Therefore, restoring the balance of intestinal microbiota has become a potential treatment strategy for epilepsy (Türay et al., 2023).

Probiotics are live microorganisms, including bacteria and yeasts, that are beneficial for human health (Kim et al., 2019). Zhang et al. (2022) demonstrated that probiotics effectively normalize the gut microbiota structure in patients with epilepsy, reduce intestinal mucosal permeability, enhance immune function, and improve treatment outcomes. Similarly, Gómez-Eguílaz et al. (2018) reported that probiotic supplementation significantly reduced seizure frequency and improved the quality of life in patients with DRE. Accumulating evidence indicates that probiotics reduce seizure frequency and severity, enhance cerebral antioxidant capacity, improve cognitive function (Bagheri et al., 2019), and regulate anxiety and depression (Aygun et al., 2022). Long-term probiotic administration also alleviates neuroinflammation and oxidative stress, shortens seizure duration, and exerts neuroprotective effects. Researchers have also noted that long-term probiotic supplementation during childhood effectively prevents epilepsy in susceptible individuals (Kilinc et al., 2021). These findings provide preliminary evidence for the use of probiotics as a complementary treatment for epilepsy. However, existing studies exhibit significant heterogeneity and lack systematic evidence synthesis. This limits our comprehensive and in-depth understanding of the role of probiotics in epilepsy treatment, hindering their rational application and promotion in clinical practice.

Therefore, this study aims to evaluate the differences in clinical efficacy and safety between probiotic supplementation combined with AEDs and AEDs alone in patients with epilepsy. We will conduct a meta-analysis of high-quality randomized controlled trials (RCTs) to provide more reliable evidence regarding the role of probiotics in epilepsy management. The primary outcomes will include the clinical efficacy rate, along with secondary outcomes such as seizure characteristics, EEG relative power across frequency bands, intestinal barrier function markers, and the total adverse reaction rate. This comprehensive analysis will enhance our understanding of the potential benefits and safety of incorporating probiotics into the treatment regimen for epilepsy.

## 2 Methods

**Figure 1:**
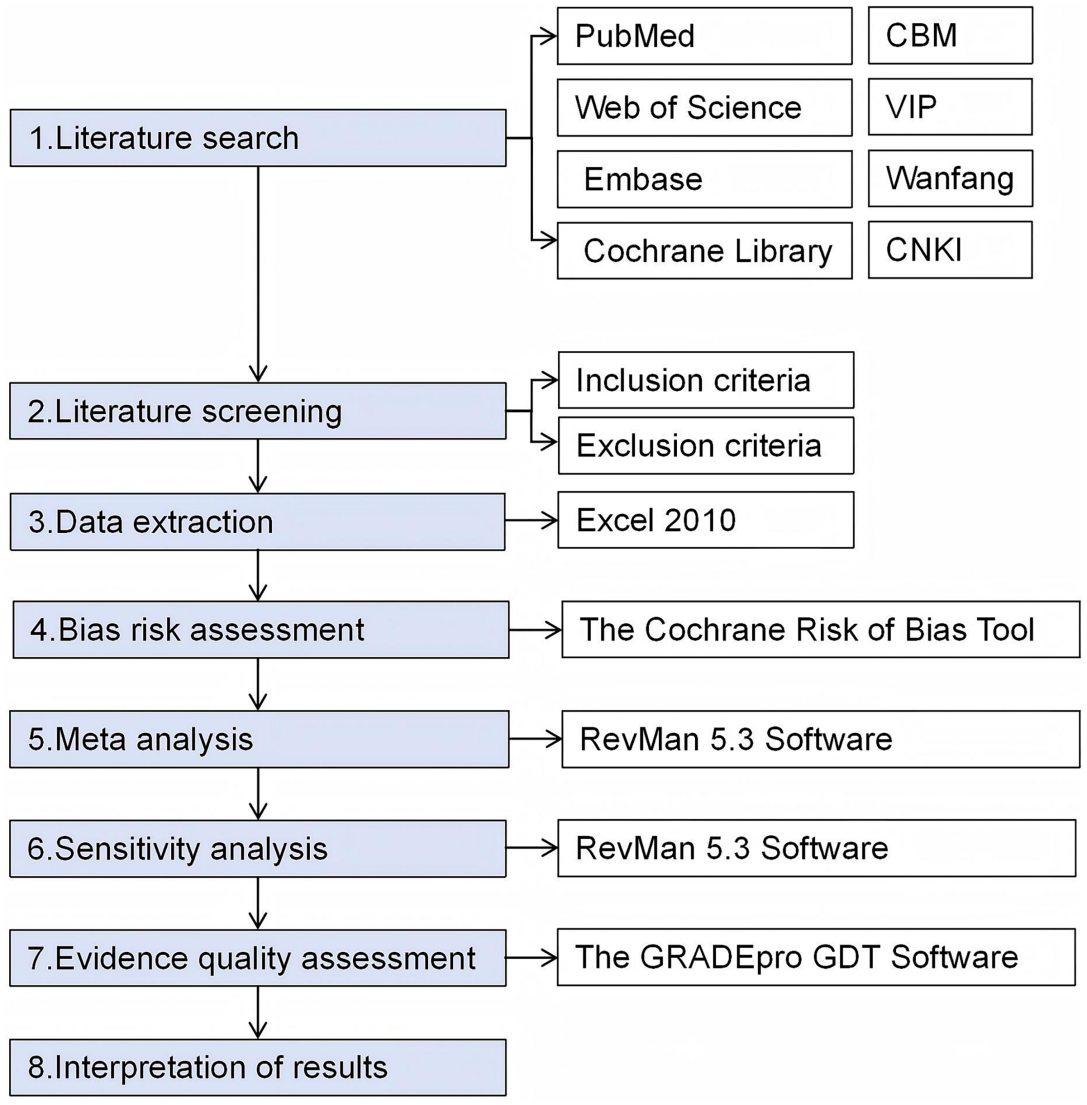
Research process flowchart.

This study adhered to the Preferred Reporting Items for Systematic Reviews and Meta-Analyses (PRISMA) guidelines and was registered with PROSPERO (registration number: 420251079172). The specific steps of the study are shown in Figure 1.

### 2.1 Literature search

We searched four English databases (PubMed, Embase, Cochrane Library, and Web of Science) and four Chinese databases (CNKI, Wanfang, VIP, and CBM) for relevant studies published up to May 31, 2025. The search was restricted to the “title/abstract” field. The English search strategy was formulated as follows: (Probiotic OR Probiotics OR Bifidobacterium OR Bifidobacteria OR Bacillus bifida OR Yeast OR *Saccharomyces cerevisiae* OR Saccharomyces italicus OR Saccharomyces oviformis OR S cerevisiae OR *S. cerevisiae* OR Saccharomyces uvarum var. melibiosus OR *Candida robusta* OR Saccharomyces capensis OR *Lactobacillus acidophilus* OR *Lactobacillus amylovorus* OR Lactobacill OR lactic acid bacteria OR *Clostridium butyricum* OR Bacillus OR Natto Bacteria OR Streptococcus thermophiles OR Enterococcus) AND (Epilepsy OR Epilepsies OR Seizure Disorder OR Seizure Disorders). The Chinese search query was constructed as follows: (益生菌 OR 益生元 OR 合生元 OR 微生态 OR 双歧杆菌 OR 酵母菌 OR 嗜酸乳杆菌 OR 乳酸菌 OR 酪酸梭菌 OR 丁酸梭菌 OR 芽孢杆菌 OR 枯草杆菌 OR 链球菌 OR 肠球菌 OR 放线菌) AND (癫痫 OR 痫证 OR 癫疯 OR 癫风 OR 癫病). No additional restrictions were applied.

### 2.2 Inclusion criteria and exclusion criteria

The inclusion criteria were as follows: (1) Study type: RCTs. (2) Study population: Patients meeting the diagnostic criteria outlined in the “Clinical Practice Guidelines: Epilepsy Volume” (Chinese Medical Association, 2007), “ILAE Official Report: A Practical Clinical Definition of Epilepsy” (Fisher et al., 2014), “A Definition and Classification of Status Epilepticus: Report of the ILAE Task Force on Classification of Status Epilepticus” (Trinka et al., 2015), or the “Expert Consensus on the Diagnosis and Treatment of Epilepsy in Children with Cerebral Palsy” (Chinese Pediatric Society, 2017). (3) Intervention and comparison: The control group received only AED treatments such as carbamazepine, oxcarbazepine, levetiracetam, sodium valproate, and lamotrigine, whereas the treatment group received probiotics based on the control group. (4) Outcome measures: The primary efficacy outcome was the clinical efficacy rate (Zhang et al., 2022), defined as the proportion of patients who experienced a ≥50% reduction in seizure frequency after treatment relative to the total number of participants. Secondary efficacy outcomes included seizure characteristics (seizure frequency, seizure duration, and interictal epileptiform discharges [IEDs]), EEG relative power across frequency bands (δ, θ, α, and β), and intestinal barrier function markers (serum diamine oxidase [DAO] and D-lactate [D-LAC] levels). The safety outcome was the total adverse reaction rate.

The exclusion criteria were as follows: (1) studies with duplicate publications; (2) animal studies, retrospective studies, and case reports; (3) studies lacking baseline data; and (4) studies with unclear key data.

### 2.3 Literature screening

All studies were imported into EndNote X9 for initial deduplication, which was verified through cross-validation of titles, authors, journal metadata (volume/issue), and digital object identifiers. Two independent reviewers (KT and YY) screened the titles and abstracts against predefined inclusion criteria and excluded studies that were irrelevant to the research objectives. The full texts of the retained articles were evaluated to confirm their eligibility for meta-analysis. Any discrepancies between the reviewers were resolved by adjudication by a third investigator (PL).

### 2.4 Data extraction

The key characteristics and data of the included studies were documented on a Microsoft Excel 2024 spreadsheet. The extracted information included the name of the first author, publication year, sample size, proportion of males, mean age, disease duration, intervention, probiotic strain, and treatment duration. Outcome-related data included all the measurements relevant to the research objectives. KT and YY independently extracted and cross-verified the baseline and statistical data, and PL resolved any disagreements.

### 2.5 Literature quality assessment

We used the Cochrane Risk of Bias Tool in RevMan 5.3 to evaluate seven domains of methodological quality: random sequence generation, allocation concealment, blinding of participants and personnel, blinding of outcome data, incomplete outcome data, selective reporting, and other biases. Two reviewers (KT and YY) independently conducted the assessments, and PL adjudicated conflicting judgments.

### 2.6 Statistical analysis

We performed meta-analyses, sensitivity analyses, and assessments of publication bias using RevMan 5.3. The mean difference (MD) was used as the effect size for continuous variables, and the relative risk (RR) was used for dichotomous outcomes. The choice between a fixed- or random-effects model was based on the degree of statistical heterogeneity as indicated by the *I*^2^ statistic. A fixed-effects model was favored when *I*^2^ was ≤ 50%, indicating minimal heterogeneity. Conversely, a random-effects model was used when *I*^2^ surpassed 50%, indicating substantial heterogeneity across studies. Statistical significance was defined as *p* < 0.05. To assess the robustness of the pooled results, we performed a sensitivity analysis using the leave-one-out method. In this procedure, each individual study was sequentially omitted from the meta-analysis, and the pooled effect size was recalculated for the remaining studies. The stability of the overall results was confirmed by observing whether the effect estimates remained consistent and without substantial fluctuation across all iterations. The absence of significant changes in the magnitude or direction of the pooled effects upon the exclusion of any single study reinforces the reliability of our findings. Potential publication biases were visually assessed using funnel plots. A symmetrical distribution of data points indicated a low likelihood of bias, whereas asymmetry suggested a potential publication bias.

### 2.7 Evidence quality

We used GRADEpro GDT software to evaluate the quality of evidence comprehensively for each outcome. The evaluation considered critical dimensions including the risk of bias, inconsistency of findings, indirectness of evidence, imprecision of effect estimates, and potential publication bias. Overall, the certainty of the evidence was classified into four categories: high, moderate, low, or very low. These classifications enabled transparent and rigorous interpretation of the findings.

## 3 Results

### 3.1 Literature screening

**Figure 2:**
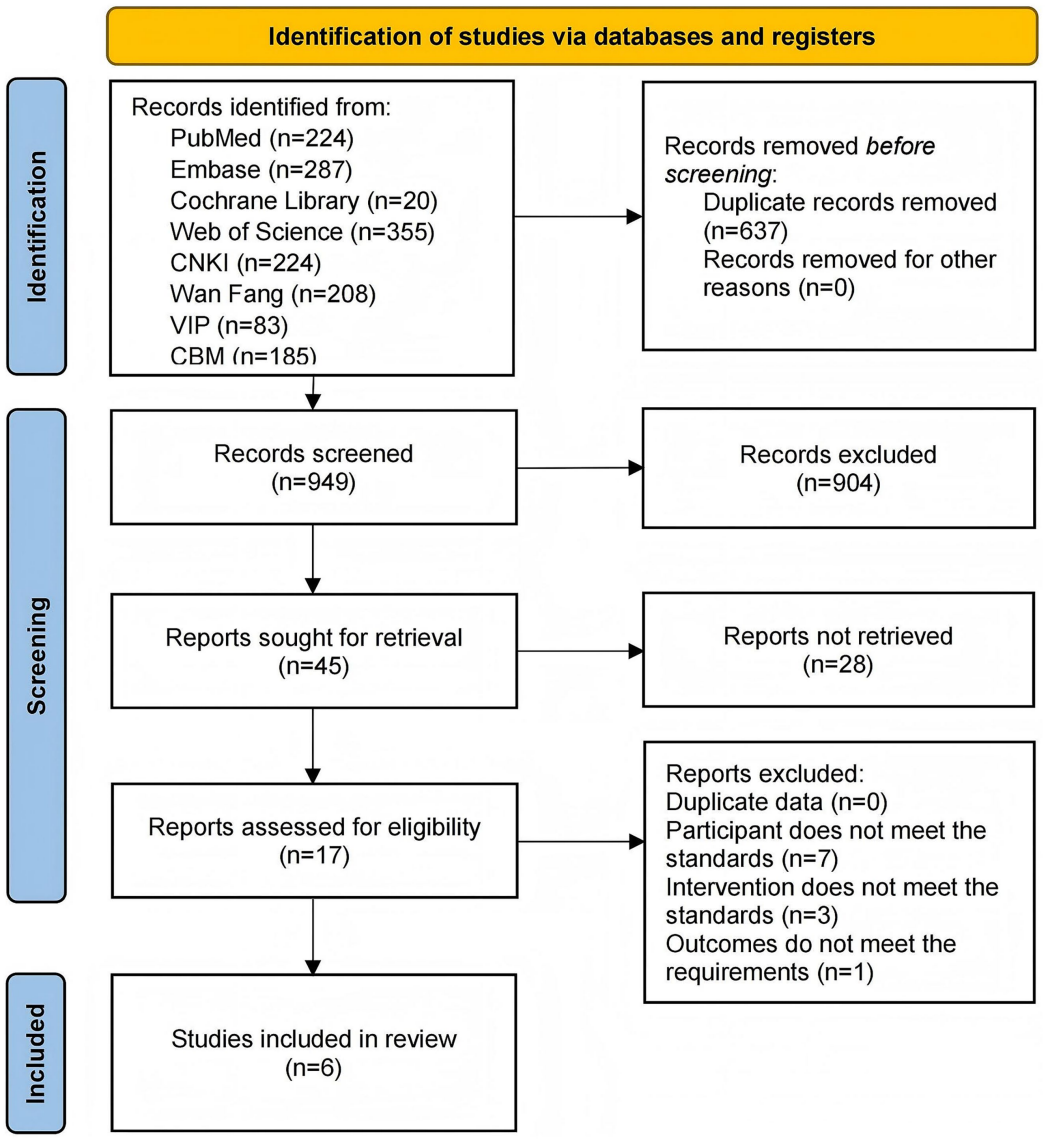
Literature screening flowchart.

A search of the eight databases yielded an initial pool of 1,586 articles. Of these, 637 articles were excluded because of duplication, and 932 articles were excluded because they were irrelevant to the study topic. After the full texts were assessed, 11 articles were excluded because they did not meet the inclusion criteria. Specifically, seven articles were non-controlled trials, three had inconsistent interventions, and one had unavailable outcome measures. Consequently, six articles (You and Wang, 2017; Jin et al., 2019; Dong et al., 2023; Tan and Zhang, 2024; Wu et al., 2024a; Yue et al., 2025) were included in the meta-analysis (Figure 2).

### 3.2 Basic characteristics of the included studies

The studies included in this review were published between 2017 and 2025. A total of 656 patients with epilepsy were included in the studies. The mean male-to-female ratio, age, disease duration, and treatment duration were 59.1%, 7.8 years, 3.2 years, and 15 weeks, respectively. Regarding AEDs, one study (Dong et al., 2023) used levetiracetam combined with lamotrigine, one (Tan and Zhang, 2024) used levetiracetam combined with sodium valproate, one (Wu et al., 2024a) administered sodium valproate or oxcarbazepine, one (Jin et al., 2019) used lamotrigine, one (You and Wang, 2017) used carbamazepine, and one (Yue et al., 2025) used levetiracetam. Regarding the bacterial strains included in the probiotic supplements, two studies (Dong et al., 2023; Tan and Zhang, 2024) included *Clostridium butyricum* and *Bifidobacterium infantis*; two (You and Wang, 2017; Jin et al., 2019) used *Saccharomyces boulardii*; one (Wu et al., 2024a) included *C. butyricum*; and one (Yue et al., 2025) employed a composite probiotic containing *Bifidobacterium longum*, *Lactobacillus acidophilus*, *Bifidobacterium bifidum*, *Bifidobacterium breve*, *B. lactis*, *Lactobacillus brevis*, *Lactobacillus delbrueckii* subsp. *bulgaricus*, *Lactobacillus casei*, *Lactobacillus helveticus*, *Lactobacillus plantarum*, *Lactobacillus reuteri*, *Lactobacillus rhamnosus*, *Lactobacillus salivarius*, *Lactococcus lactis*, *Streptococcus thermophilus*, and *B. infantis* (Table 1).

**Table 1:** Basic characteristics of included studies.

Study	Sample size	Male (%)	Age (years)	Disease duration (years)	Intervention	Comparison	Probiotics	Treatment duration (weeks)
Dong et al. (2023)	52/52	59.6	8.2	3	Combined *clostridium butyricum* and bifidobacterium powders, levetiracetam, and lamotrigine	Levetiracetam and lamotrigine	*C. butyricum* and *B. infantis*	24
Jin et al. (2019)	70/60	53.8	12.4	4.4	Saccharomyces boulardii sachets and lamotrigine	Lamotrigine	*S. boulardii*	4
Tan and Zhang (2024)	44/44	55.7	6.1	1.3	Combined *clostridium butyricum* and bifidobacterium powders, sodium valproate, and levetiracetam	Sodium valproate and levetiracetam	*C. butyricum* and *B. infantis*	12
Wu et al. (2024a)	55/55	68.2	9.3	1.2	*Clostridium butyricum* tablets and sodium valproate or oxcarbazepine	Sodium valproate or oxcarbazepine	*C. butyricum*	24
You and Wang (2017)	56/56	60.7	2.8	4.6	Saccharomyces boulardii sachets and carbamazepine	Carbamazepine	*S. boulardii*	4
Yue et al. (2025)	56/56	57.1	7.2	4.1	Probiotic complex solid beverage and levetiracetam	Levetiracetam	*S. boulardii*, *B. longum*, *L. acidophilus*, *B. bifidum*, *B. breve*, *B. lactis*, *L. brevis*, *L. delbrueckii* subsp. *bulgaricus*, *L. casei*, *L. helveticus*, *L. plantarum*, *L. reuteri*, *L. rhamnosus*, *L. salivarius*, *L. lactis*, *S. thermophilus*, and *B. infantis*.	24

### 3.3 Literature quality assessment

**Figure 3:**
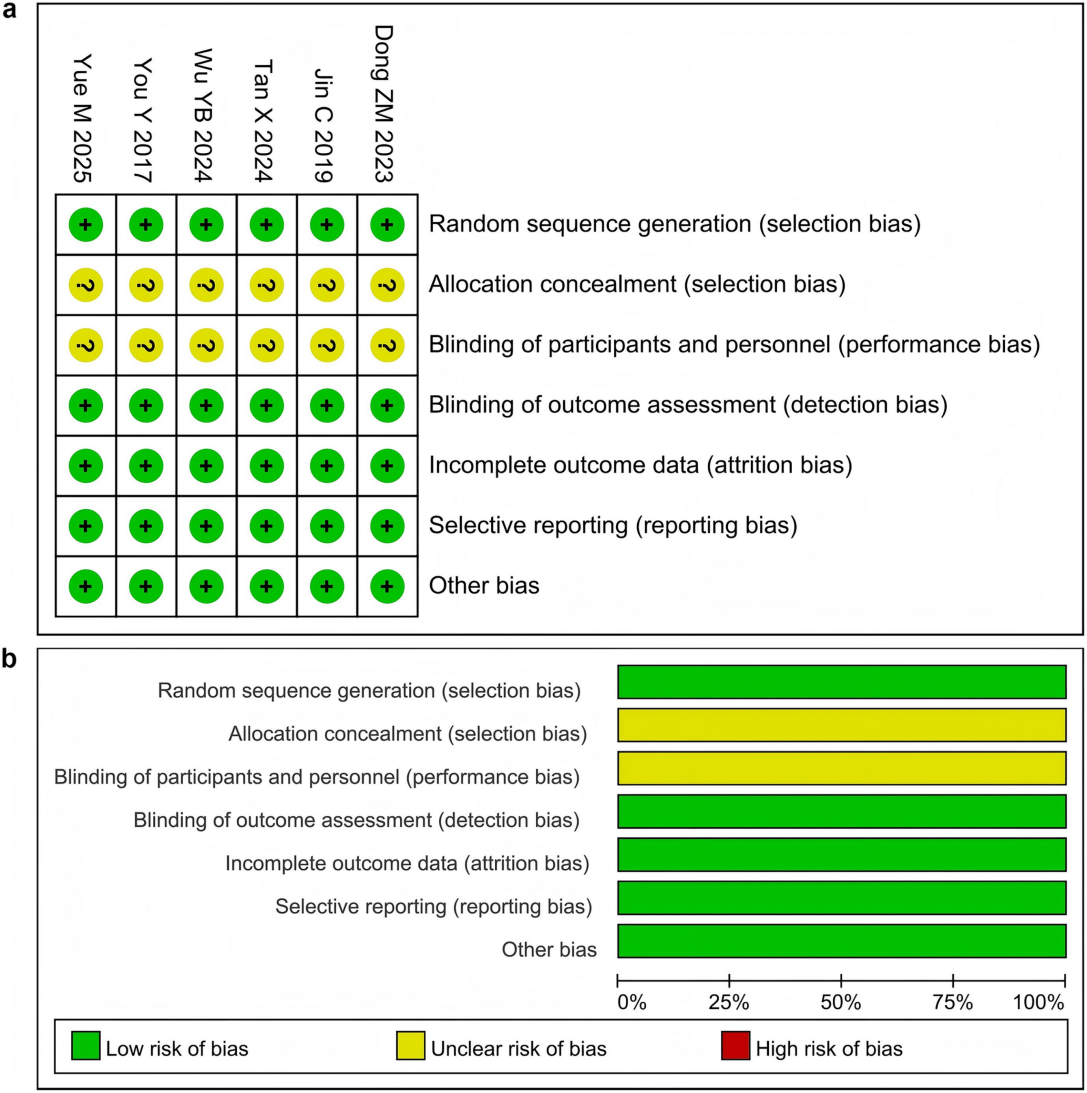
Risk of bias assessment. **(a)** traffic light plot, **(b)** summary barplot.

All six studies (You and Wang, 2017; Jin et al., 2019; Dong et al., 2023; Tan and Zhang, 2024; Wu et al., 2024a; Yue et al., 2025) in the random sequence generation assessment explicitly reported the specific methods used to generate random sequences and were judged to have a low risk of bias. However, in the allocation concealment and blinding of participants and personnel assessment, the specific implementation methods for allocation concealment were not detailed, and placebo-controlled designs were not used in the trial design to ensure a double-blind process. Therefore, all six studies (You and Wang, 2017; Jin et al., 2019; Dong et al., 2023; Tan and Zhang, 2024; Wu et al., 2024a; Yue et al., 2025) were judged to have an uncertain risk of bias. In the blinding of outcome data, incomplete outcome data, selective reporting, and other bias assessments, all studies fully reported the outcome data, with no evidence of loss to follow-up, selective reporting, or conflict of interest bias. Thus, all six studies (You and Wang, 2017; Jin et al., 2019; Dong et al., 2023; Tan and Zhang, 2024; Wu et al., 2024a; Yue et al., 2025) were judged to have a low risk of bias (Figure 3).

### 3.4 Meta-analysis

#### 3.4.1 Clinical efficacy rate

**Figure 4:**
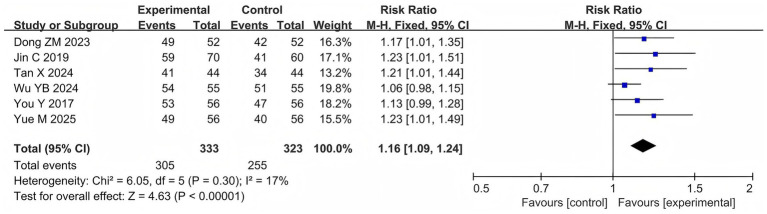
Forest plots of meta-analyses on clinical efficacy rate.

Six studies (You and Wang, 2017; Jin et al., 2019; Dong et al., 2023; Tan and Zhang, 2024; Wu et al., 2024a; Yue et al., 2025) reported the clinical efficacy rates in 656 patients. Meta-analysis revealed that the clinical efficacy rate in the probiotic combination group was significantly higher than that in the AED group (RR = 1.16, 95% confidence interval [CI] 1.09 to 1.24, *p* < 0.00001, *I*^2^ = 17%) (Figure 4).

#### 3.4.2 Epileptic seizure parameters

**Figure 5:**
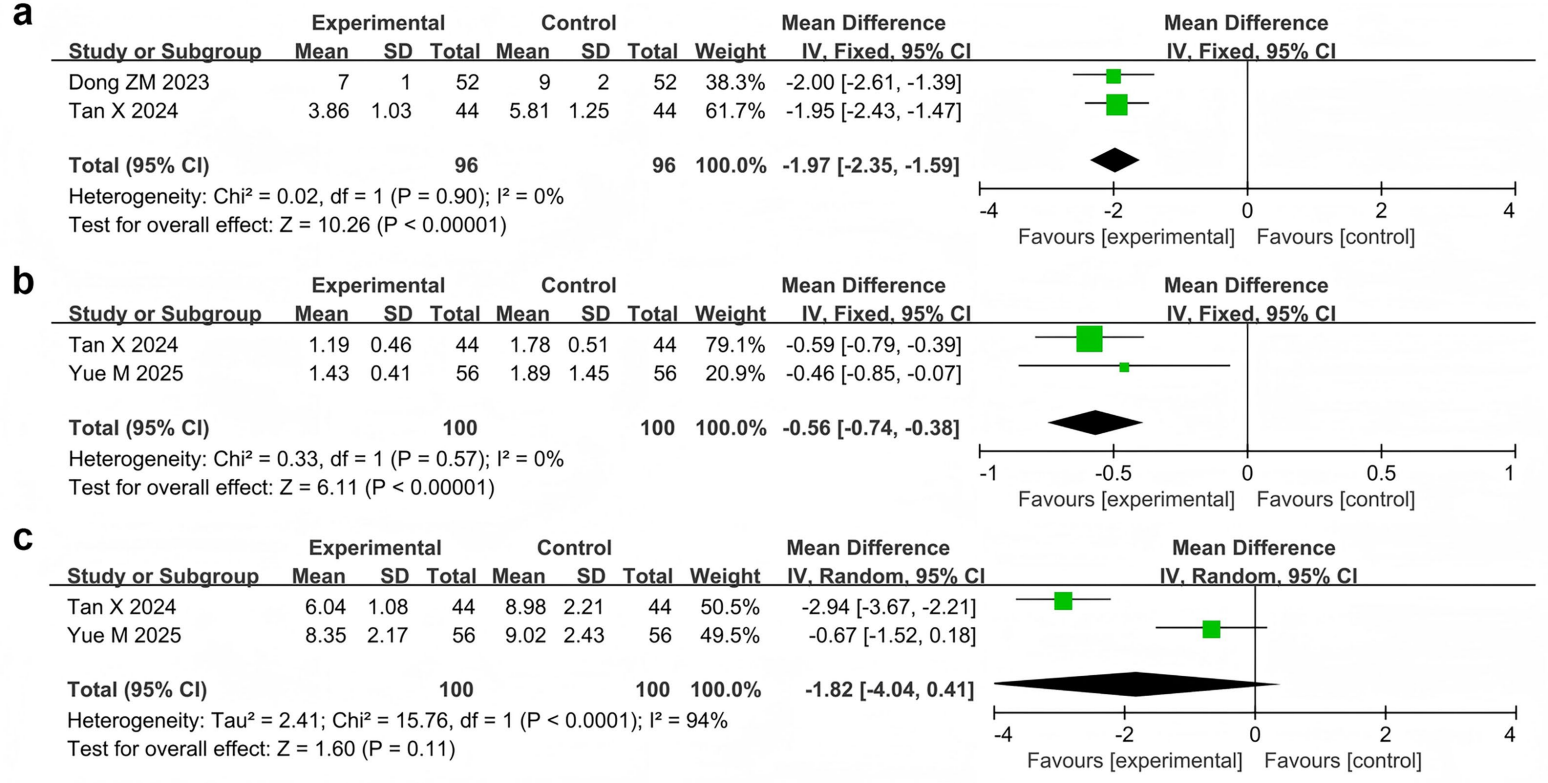
Forest plots of meta-analyses on epileptic seizure parameters: **(a)** seizure frequency, **(b)** seizure duration, **(c)** IEDs.

Two studies (Dong et al., 2023; Tan and Zhang, 2024) reported the seizure frequency in 192 patients. Meta-analysis showed that the seizure frequency in the probiotic combination group was significantly lower than that in the AED group (MD = −1.97, 95% CI −2.35 to −1.59, *p* < 0.00001, *I*^2^ = 0%) (Figure 5a).

Two studies (Tan and Zhang, 2024; Yue et al., 2025) reported seizure durations and IEDs in 200 patients. The meta-analysis showed that the duration of epilepsy was significantly shorter in the probiotic combination group than in the AED group (MD = −0.56, 95% CI -0.74 to −0.38, *p* < 0.00001, *I*^2^ = 0%) (Figure 5b). However, no significant difference in IEDs was observed between the two groups (MD = −1.82, 95% CI -4.04 to 0.41, *p* = 0.11, *I*^2^ = 94%) (Figure 5c).

#### 3.4.3 Relative power of EEG bands

**Figure 6:**
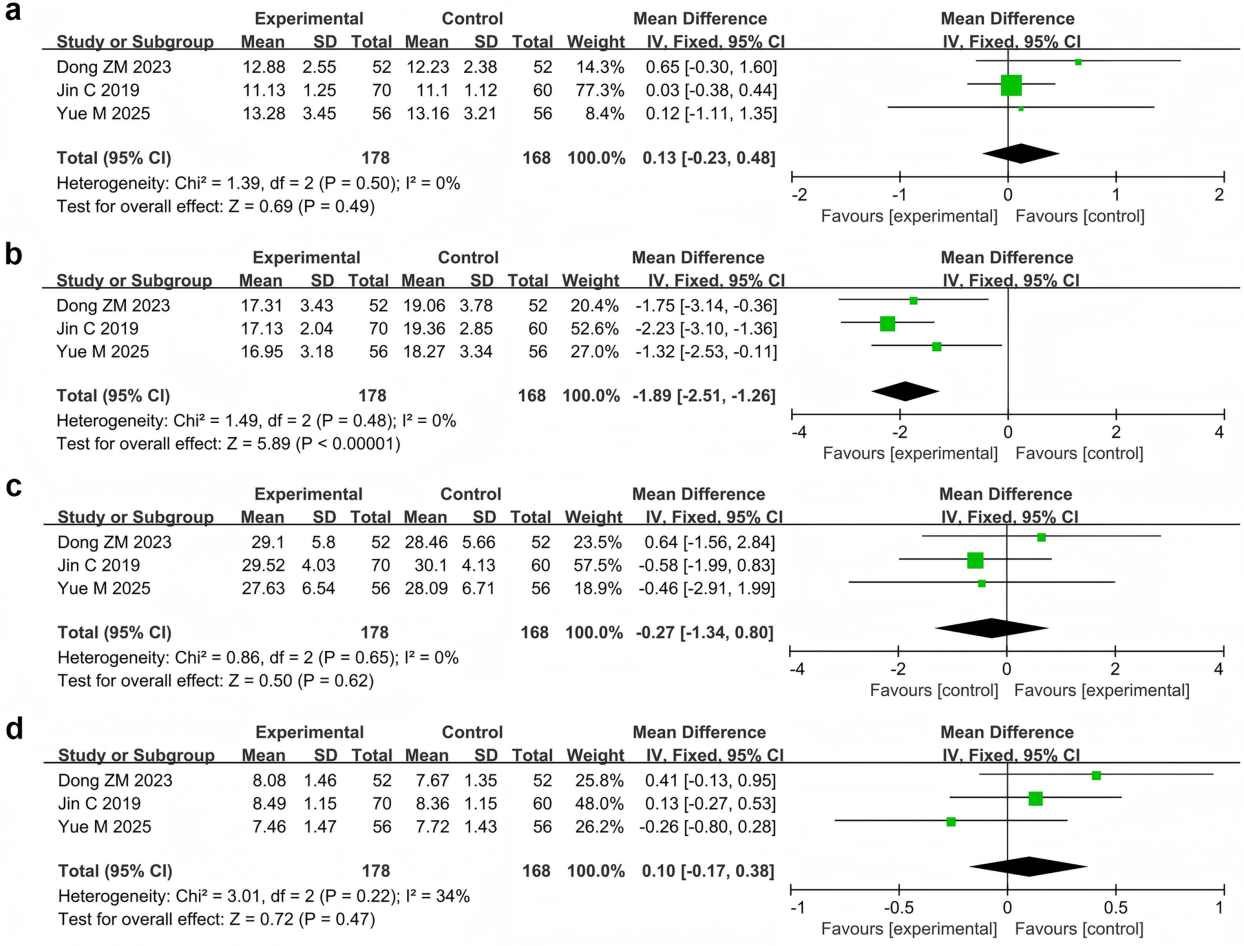
Forest plots of meta-analyses on the relative power of EEG bands: **(a)** δ band, **(b)** θ band, **(c)** α band, **(d)** β band.

Three studies (Jin et al., 2019; Dong et al., 2023; Yue et al., 2025) reported the relative power of the EEG bands in 346 patients. Meta-analysis revealed that the relative power of the θ band was significantly lower in the probiotic combination group than in the AED group (MD = −1.89, 95% CI −2.51 to −1.26, *p* < 0.00001, *I*^2^ = 0%). However, no significant differences in relative power were observed in the δ band (MD = 0.13, 95% CI −0.23 to 0.48, *p* = 0.49, *I*^2^ = 0%), *α* band (MD = −0.27, 95% CI −1.34 to 0.80, *p* = 0.62, *I*^2^ = 0%), and β band (MD = 0.10, 95% CI −0.17 to 0.38, *p* = 0.47, *I*^2^ = 0%) compared with those in the AED group (Figure 6).

#### 3.4.4 Markers of intestinal barrier function

**Figure 7:**
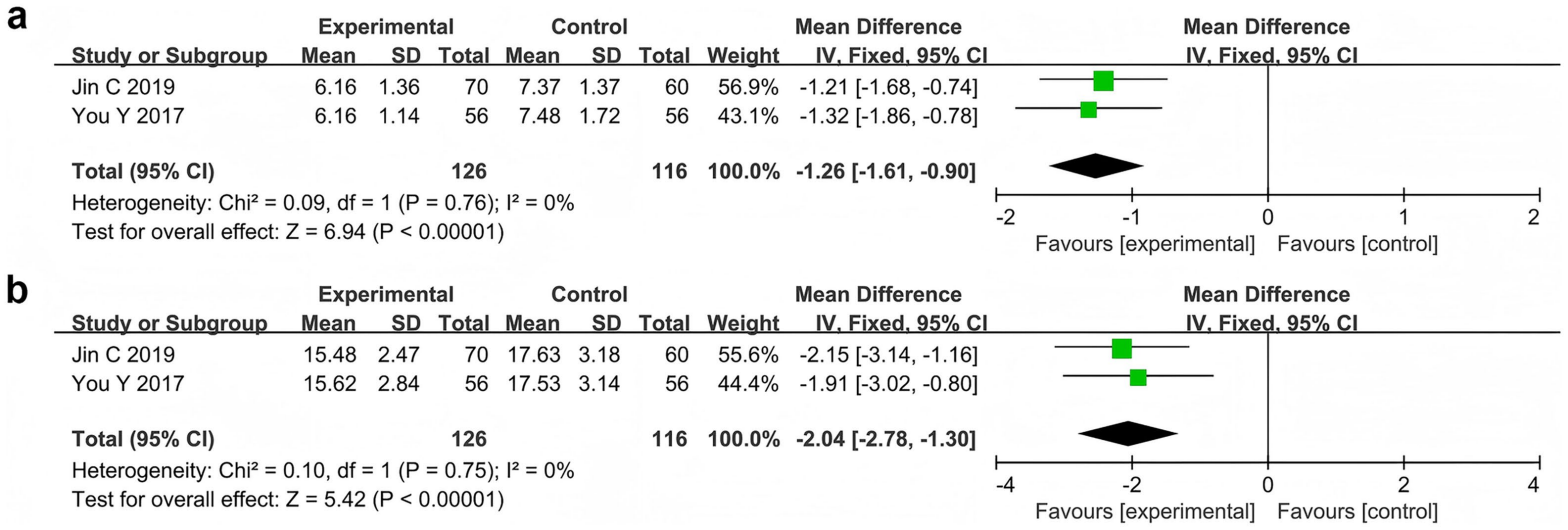
Forest plots of meta-analyses on markers of intestinal barrier function: **(a)** Serum DAO levels and **(b)** serum D-LAC levels.

Two studies (You and Wang, 2017; Jin et al., 2019) reported the serum DAO and D-LAC levels in 242 patients. The meta-analysis showed that the serum DAO levels (MD = −1.26, 95% CI −1.61 to −0.90, *p* < 0.00001, *I*^2^ = 0%) and serum D-LAC levels (MD = −2.04, 95% CI −2.78 to −1.30, *p* < 0.00001, *I*^2^ = 0%) were significantly lower in the probiotic combination group than in the AED group (Figure 7).

#### 3.4.5 Adverse reaction rate

**Figure 8:**
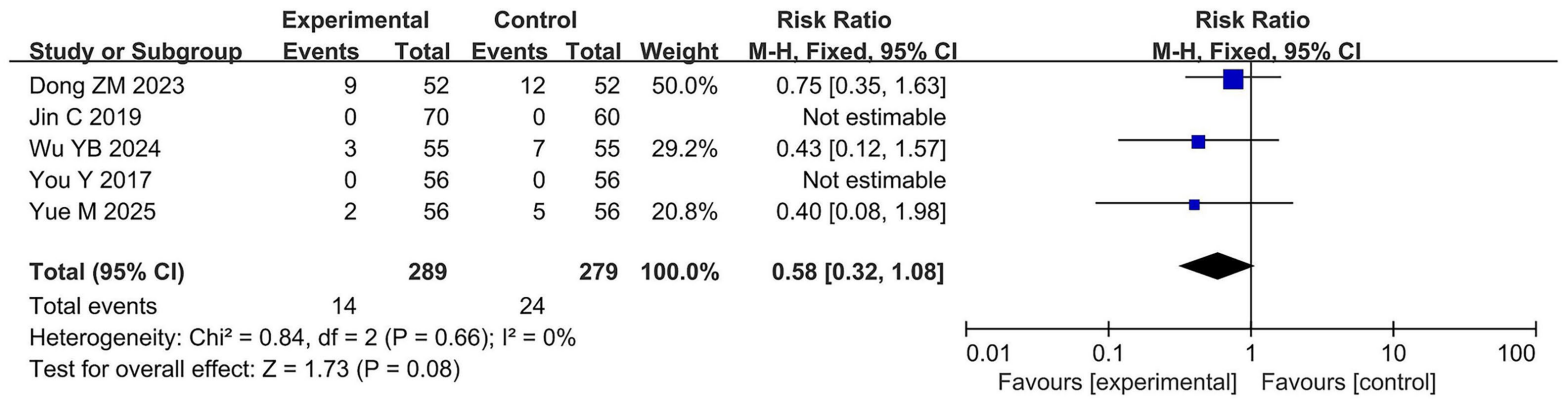
Forest plots of meta-analyses on adverse reaction rate.

Five studies (You and Wang, 2017; Jin et al., 2019; Dong et al., 2023; Wu et al., 2024a; Yue et al., 2025) reported the total adverse reaction rate involving 568 patients with 38 adverse events. Specifically, the probiotic combination group reported 14 adverse events, including four cases of dizziness, three of somnolence, two of mood abnormalities, two of rash, two of nausea and vomiting, and one of fatigue. The AED group reported 24 adverse reactions, including six cases of dizziness, four of rash, three of somnolence, three of nausea and vomiting, two of mood abnormalities, two of fatigue, and one each of fever, headache, apathy, and abdominal pain and diarrhea. Meta-analysis showed no significant difference in the total adverse reaction rate between the probiotic combination and AED groups (RR = 0.58, 95% CI 0.32 to 1.08, *p* = 0.08, *I*^2^ = 0%) (Figure 8).

### 3.5 Sensitivity analysis

A comprehensive sensitivity analysis was conducted to evaluate the robustness of the study findings. The results demonstrated that the outcomes pertaining to clinical efficacy rate, relative power of different EEG frequency bands (including δ, θ, α, and β bands), and total adverse reaction rate remained consistent and reliable under varying analytical conditions. This indicates that these particular metrics are robust and not significantly influenced by potential variations in study design or data collection methods. However, because seizure frequency, seizure duration, IEDs, and serum DAO and D-LAC levels were only included in two studies, a sensitivity analysis was not performed.

### 3.6 Publication bias

**Figure 9:**
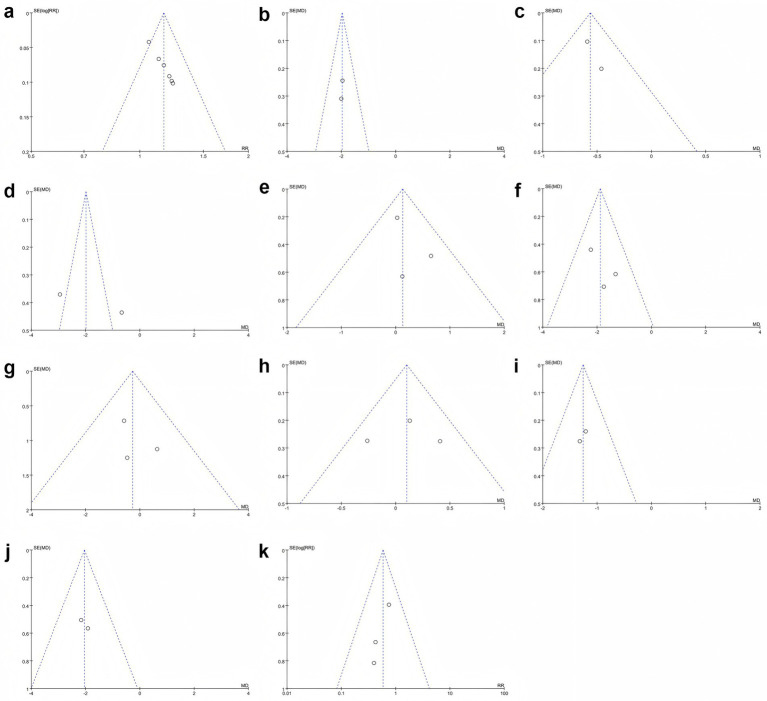
Funnel plots of publication bias **(a–k)**. **(a)** Clinical efficacy rate, **(b)** Seizure frequency, **(c)** Seizure duration, **(d)** IEDs, **(e)** Relative power of EEG δ band, **(f)** Relative power of EEG θ band, **(g)** Relative power of EEG α band, **(h)** Relative power of EEG β band, **(i)** Serum DAO levels, **(j)** Serum D-LAC levels, **(k)** Adverse reaction rate.

Funnel plots of clinical efficacy rate, seizure frequency, seizure duration, relative power of the EEG β band, and serum DAO and D-LAC levels showed roughly symmetrical scatter plots, suggesting no obvious publication bias. In contrast, the funnel plots for IEDs; the relative power of the EEG δ, θ, and α bands; and the total adverse reaction rate showed asymmetric scatter plots, suggesting potential publication bias (Figure 9).

### 3.7 Quality of evidence

The quality of evidence for clinical efficacy was moderate. The quality of evidence for seizure frequency, seizure duration, relative power of EEG β band, and serum DAO and D-LAC levels was low. Moreover, the quality of evidence for IEDs; relative power of EEG δ, θ, and α bands; and total adverse reaction rate was very low (Table 2).

**Table 2:** Quality of evidence.

Outcome	Risk of bias	Inconsistency	Indirectness	Imprecision	Publication bias	Quality of evidence
Clinical efficacy rate	Serious	None	None	None	None	Moderate
Seizure frequency	Serious	None	None	Serious	None	Low
Seizure duration	Serious	None	None	Serious	None	Low
IEDs	Serious	Very serious	None	Serious	Serious	Very Low
Relative power of EEG δ band	Serious	None	None	Serious	Serious	Very Low
Relative power of EEG θ band	Serious	None	None	Serious	Serious	Very Low
Relative power of EEG α band	Serious	None	None	Serious	Serious	Very Low
Relative power of EEG β band	Serious	None	None	Serious	None	Low
Serum DAO levels	Serious	None	None	Serious	None	Low
Serum D-LAC levels	Serious	None	None	Serious	None	Low
Adverse reaction rate	Serious	None	None	Serious	Serious	Very Low

## 4 Discussion

### 4.1 Main findings

To the best of our knowledge, this is the first meta-analysis to assess the efficacy and safety of probiotic combination therapy with AEDs for epilepsy. Our results showed that, compared with AEDs, probiotic combination therapy significantly improved clinical efficacy rate, reduced seizure frequency and duration, as well as the relative power of EEG θ band and serum DAO and D-LAC levels. Furthermore, the use of probiotics did not increase the incidence of adverse events.

### 4.2 Therapeutic efficacy

This study found that the probiotic combination therapy significantly improved the clinical efficacy rate, reduced seizure frequency, and decreased seizure duration. These results are consistent with those of previous studies (Zhang et al., 2022; Tan and Zhang, 2024; Yue et al., 2025). Although IEDs are the gold standard for seizure assessment (Guida et al., 2015), our results suggest that the probiotic combination therapy had no significant effect on IEDs. This may be related to the fact that IEDs have high specificity but low sensitivity (Kienitz et al., 2025). Studies have indicated that approximately 50% of patients with epilepsy exhibit IEDs on their initial EEG (Gregory, 1997), whereas approximately 10% show no IEDs even after prolonged monitoring (Salinsky et al., 1987). In addition, the IED latency periods vary among patients with different types of epilepsy. For example, patients with late-onset epilepsy or those who have not recently experienced seizures may require longer monitoring periods (Liu et al., 2023). Despite these confounding factors, probiotics have shown the potential to improve epilepsy. Ishii et al. (2024) found that oral administration of *Bifidobacterium breve* strain A1 significantly reduced seizure scores in a pentylenetetrazole-induced kindling mouse model. Shariatmadari et al. (2024) reported that probiotic supplementation reduces seizure frequency in children with DRE and alleviates the burden on their caregivers. Recurrent seizures and long-term medication use often lead to cognitive decline, and probiotic supplementation may ameliorate this decline (Wu et al., 2025). Jin et al. (2019) found that cognitive function scores significantly improved in children treated with *S. boulardii* combined with lamotrigine. Similarly, Yue et al. (2025) reported that patients’ Mini-Mental State Examination scores increased after probiotic combination therapy, further confirming its protective effect on cognitive function. Wang et al. (2022) found that probiotics not only control seizures and improve cognition but also alleviate anxiety and depression, enhancing patients’ quality of life. In summary, probiotics are beneficial for patients with epilepsy cognitively and emotionally.

This meta-analysis revealed that probiotic combination therapy significantly reduced the relative power of the EEG θ band. Increased θ band relative power is commonly observed in patients with epilepsy and is associated with excessive brain excitability and cognitive dysfunction (Glaba et al., 2020). The reduction of relative power in the θ band by probiotics is consistent with results showing that probiotics reduce the frequency and duration of epileptic seizures (Dong et al., 2023). This suggests that probiotics play a role in ameliorating abnormal brain excitation. However, our analysis indicated that probiotic combination therapy had no significant effect on the relative power of the δ, α, or β bands. This finding is inconsistent with that of previous studies (Zhang et al., 2022). The δ band primarily appears during deep sleep but also occurs during wakefulness if normal brain rhythms are disrupted. In some pediatric patients with epilepsy, particularly those with cognitive dysfunction or brain injury, the δ band increases (Nair et al., 2014). Moreover, the δ band serves as a diagnostic indicator for assessing recurrent epilepsy after surgery. At rest, α and β bands are primarily evident (Bencze et al., 2025). During the interictal periods in patients with epilepsy, the rhythms of these two bands slow down, weaken, or disappear, indicating brain lesions or functional abnormalities (Rezazadeh et al., 2023; Garnica-Agudelo et al., 2025). The results of probiotic combination therapy for EEG in patients with epilepsy are controversial and may be related to differences in instruments, variations in parameter settings, significant differences among patients, or small sample sizes. These factors lead to a high heterogeneity in the study results. Therefore, future evaluations of EEG efficacy in patients with epilepsy require more detailed and rigorous controls.

Serum DAO and D-LAC levels are reliable biomarkers for assessing intestinal barrier integrity (You and Wang, 2017; Jin et al., 2019). Serum DAO is primarily present in the villous epithelium of the intestinal mucosa, and its levels increase when the barrier is damaged (Yuan et al., 2025). Serum D-LAC is a fermentation product of intestinal bacteria with minimal absorption under normal barrier function. When the barrier is damaged, the amount entering the bloodstream increases (Remund et al., 2023). This study demonstrated that probiotic combination therapy significantly reduced serum DAO and D-LAC levels, indicating that probiotics play a role in improving intestinal barrier function.

### 4.3 Potential mechanisms

Probiotics exert antiepileptic effects through multiple mechanisms (Iannone et al., 2022), with the primary mechanism being the regulation of the gut microbiota. Probiotics competitively inhibit the growth of harmful bacteria while promoting the growth of beneficial bacteria (García Mansilla et al., 2024). The gut microbiota regulates the synthesis and metabolism of key neurotransmitters such as gamma-aminobutyric acid (GABA) and 5-hydroxytryptamine (5-HT) (Chen et al., 2022). GABA, the principal central inhibitory neurotransmitter, is reduced before seizures (Belelli et al., 2025). Patients with epilepsy have reduced numbers of probiotics that regulate GABA, such as *Bifidobacterium* and *Lactobacilli*, in their gut (Sabouri et al., 2021). Supplementation with these probiotics increases GABA levels in the brain, inhibits abnormal neuronal discharge, and reduces epileptic seizures (Eor et al., 2021). 5-HT indirectly exerts antiepileptic activity by modulating neural network excitability, and the gut microbiota influences 5-HT synthesis by regulating tryptophan metabolism (Peng et al., 2023). Moreover, research has shown that combining probiotics and pregabalin provides antioxidant neuroprotection and reverses neuronal loss (Ali et al., 2025). This combination prevents pathological neurodegenerative diseases and suppresses neuropsychiatric comorbidities in generalized epilepsy models. The interaction between probiotics and the gut microbiota not only influences the gut-brain axis but also reduces neuroinflammation and oxidative stress (Shirzadi et al., 2025). Specifically, probiotic supplementation restored levels of the oxidant thiobarbituric acid reactive substances (TOS) and disulfides, as well as the antioxidants natural thiols and total thiols, in a rat model of pentazocine-induced seizures (Kilinc et al., 2021). Additionally, probiotics suppress proinflammatory cytokine production, alleviate neuroinflammatory damage (Shirzadi et al., 2025), and produce metabolites with neuroprotective effects, such as short-chain fatty acids (SCFAs) (Yan et al., 2025). Therefore, probiotic combination therapy may indirectly improve seizures by reshaping the gut microbiota, influencing neurotransmitter synthesis and metabolism, and reducing inflammatory responses.

In addition to an imbalance in the intestinal microbiota, damage to the intestinal barrier function is a key factor that contributes to the progression of epilepsy (Bian and Shao, 2024). Probiotics improve the intestinal barrier function through several mechanisms. First, probiotics regulate the balance of the intestinal microbiota, enhance the integrity of the intestinal mucosal barrier, promote the proliferation and repair of intestinal mucosal cells, and increase the expression of tight junction proteins (Rahman et al., 2018; Wu et al., 2024b; Zhu et al., 2024). Second, probiotics suppress intestinal inflammatory responses, reduce intestinal permeability, and mitigate inflammation-induced damage to the intestinal barrier (Cao et al., 2023). Third, probiotics produce metabolic byproducts such as SCFAs, which provide energy to intestinal mucosal cells and promote repair and regeneration of the intestinal mucosa (Hays et al., 2024). In summary, probiotic supplementation alleviates the damage to the intestinal barrier caused by dysbiosis, controlling the onset and progression of epilepsy.

Gut microbiota is closely associated with the immune system (Zhou et al., 2020). Supplementation with probiotics can reshape the gut microenvironment, regulate the immune system, and reduce the frequency of epileptic seizures (Mirzababaei et al., 2025). Probiotics treat diseases by inhibiting the release of proinflammatory factors and regulating immune cell functions (de Jesus et al., 2024). Dong et al. (2023) confirmed that probiotic combination therapy significantly reduced levels of inflammatory factors, such as interleukin-6 and tumor necrosis factor-*α*, alleviating neuroinflammatory damage. Yue et al. (2025) found that a novel composite probiotic combined with levetiracetam treatment increased CD3+ and CD4+ levels and the CD4+/CD8+ ratio. This enhanced the immune regulatory capacity of patients and inhibited inflammation-related seizures. Improvement in immune markers enhances immunity, reduces infection-triggered seizures, and alleviates epilepsy symptoms (Vega García et al., 2025).

### 4.4 Safety analysis

This meta-analysis found that the incidence of adverse events was 5% (14/289) and 9% (24/279) in the probiotic combination and AED groups, respectively. No statistically significant difference was observed in the total incidence of adverse events between the two groups, indicating that the probiotic and AED combination was safe. The observed adverse reactions in the probiotic combination group included 4 cases of dizziness, 3 cases of somnolence, 2 cases of mood abnormalities, 2 cases of rash, 2 cases of nausea and vomiting, and 1 case of fatigue. Studies (Zeng et al., 2010; Dang et al., 2021) have noted that the most common adverse events associated with AED monotherapy include anxiety, depression, insomnia, headaches, gastrointestinal disorders, loss of appetite, nausea, weight gain, fatigue, tremors, and rashes. Adverse reactions caused by probiotics (e.g., dizziness, rash, abdominal pain, diarrhea, nausea, and vomiting) are usually mild and reversible (Xie et al., 2022; Kong et al., 2024). Therefore, the adverse reactions observed in the probiotic combination group were likely attributable to AEDs. A previous meta-analysis showed that probiotic combination therapy did not significantly increase the risk of adverse reactions (Xie et al., 2022). This finding indicates that probiotics have potential applications as safe biological agents in epilepsy combination therapy.

### 4.5 Research gaps and contributions

Although previous studies have investigated the potential role of probiotics in epilepsy, the existing evidence remains fragmented and lacks systematic integration. Earlier research includes observational analyses, small clinical trials, and mechanistic explorations, but these studies have focused on isolated probiotic strains or individual clinical outcomes and have not evaluated their overall therapeutic value. Differences in study design, probiotic formulations, and outcome indicators have made it difficult to reach consistent conclusions or to translate research findings into clinical practice. Importantly, no published systematic review has provided a comprehensive evaluation of the efficacy and safety of probiotic combination therapy with antiepileptic drugs. As a result, it is still unclear whether probiotics confer additional benefits beyond standard treatment, which patient groups may respond most favorably, and which clinical or biological markers show meaningful changes following supplementation.

The present meta-analysis helps address these research gaps in several ways. First, it synthesizes data from six randomized controlled trials, offering the most up-to-date and comprehensive assessment of the therapeutic effects of probiotic combination therapy in epilepsy. Second, this study integrates multiple outcome dimensions, including seizure activity, electroencephalographic parameters, and intestinal barrier biomarkers, thereby providing a more complete understanding of the potential pathways through which probiotics may influence neurological outcomes. Third, by comparing our findings with previous clinical and mechanistic evidence, this work expands and refines existing knowledge and underscores the gut–brain axis as a plausible therapeutic target in epilepsy management. Finally, through rigorous risk-of-bias assessment and evidence grading, the study clarifies the strength and limitations of the available evidence and provides well-defined directions for future research.

### 4.6 Limitations and outlook

Although this meta-analysis provides evidence for the efficacy and safety of probiotic combination therapy for epilepsy, it has some limitations. First, the small number of included studies and relatively small sample sizes may have led to selection bias in the results and affected the generalizability of the conclusions. Second, the included studies had inconsistent methodological quality with insufficient details on aspects such as allocation concealment and blinding. This reduced the reliability and robustness of the results. Additionally, all participants in the included studies were from the Chinese population, which limited the generalizability of the findings. Furthermore, the probiotic formulations used in these studies varied significantly in terms of the type, dose, and duration of treatment. This increases the heterogeneity between studies and complicates the pooling and analysis of the results. Finally, no follow-up reports have been made, and evidence regarding the long-term efficacy and safety of probiotic combination therapy is insufficient.

Therefore, future studies should expand the scale and scope of this study. They should conduct multicenter, large-sample, RCTs that include patients with epilepsy from different regions and with different ethnicities and clinical characteristics. This will allow a comprehensive assessment of the efficacy and safety of probiotic combination therapy in different populations. Strict adherence to the methodological principles of RCTs, including measures such as blinding and random allocation concealment, is necessary to reduce bias and improve the quality of evidence. Long-term follow-up studies should be conducted to fully assess the long-term efficacy and safety of probiotic combination therapy and provide more comprehensive references for clinical application.

## 5 Conclusion

Compared with AEDs, probiotic combination therapy improved seizure frequency, relative power of certain EEG frequency bands, and intestinal barrier function and did not increase adverse events. These results highlight the potential of probiotics as a complementary treatment for epilepsy. However, owing to the small sample size and low methodological quality of the studies, these results need to be validated by large, multicenter, long-term, high-quality clinical studies.

## Data Availability

The original contributions presented in the study are included in the article/supplementary material, further inquiries can be directed to the corresponding authors.
